# Evaluation of whole-genome sequencing for outbreak detection of Verotoxigenic *Escherichia coli* O157:H7 from the Canadian perspective

**DOI:** 10.1186/s12864-018-5243-3

**Published:** 2018-12-04

**Authors:** Jillian Rumore, Lorelee Tschetter, Ashley Kearney, Rima Kandar, Rachel McCormick, Matthew Walker, Christy-Lynn Peterson, Aleisha Reimer, Celine Nadon

**Affiliations:** 1 Division of Enteric Diseases, National Microbiology Laboratory, Public Health Agency of Canada, Winnipeg, MB Canada; 2 Outbreak Management Division, Centre for Foodborne, Environmental and Zoonotic Infectious Diseases, Public Health Agency of Canada, Guelph, ON Canada; 3 Department of Medical Microbiology and Infectious Diseases, University of Manitoba, Winnipeg, MB Canada

**Keywords:** VTEC, *Escherichia coli*, O157:H7, Whole genome sequencing, Outbreak, wgMLST, SNVPhyl

## Abstract

**Background:**

Rapid and accurate identification of Verotoxigenic *Escherichia coli* (VTEC) O157:H7 is dependent on well-established, standardized and highly discriminatory typing methods. Currently, conventional subtyping tests for foodborne bacterial pathogen surveillance are rapidly being replaced with whole-genome sequencing (WGS) in public health laboratories. The capacity of WGS to revolutionize global foodborne disease surveillance has positioned this tool to become the new gold standard; however, to ensure evidence standards for public health decision making can still be achieved, the performance of WGS must be thoroughly validated against current gold standard methods prior to implementation. Here we aim to verify the performance of WGS in comparison to pulsed-field gel electrophoresis (PFGE) and multiple-locus variable-number tandem repeat analysis (MLVA) for eight retrospective outbreaks of VTEC O157:H7 from the Canadian perspective. Since real-time implementation and routine use of WGS in public health laboratories is highly reliant on standardized data analysis tools, we also provide a comparative analysis of two popular methodologies for WGS analyses; an in-house developed single nucleotide variant phylogenomics (SNVPhyl) pipeline and the BioNumerics whole genome multilocus sequence typing (wgMLST) tool. To provide a useful and consistent starting point for examining laboratory-based surveillance data for VTEC O157:H7 in Canada, we also aim to describe the number of genetic differences observed among outbreak-associated isolates.

**Results:**

WGS provided enhanced resolution over traditional subtyping methods, and accurately distinguished outbreak-related isolates from non-outbreak related isolates with high epidemiological concordance. WGS also illuminated potential linkages between sporadic cases of illness and contaminated food, and isolates spanning multiple years. The topologies generated by SNVPhyl and wgMLST were highly congruent with strong statistical support. Few genetic differences were observed among outbreak-related isolates (≤5 SNVs/ < 10 wgMLST alleles) unless the outbreak was suspected to be multi-strain.

**Conclusions:**

This study validates the superiority of WGS and indicates the BioNumerics wgMLST schema is suitable for surveillance and cluster detection of VTEC O157:H7. These findings will provide a useful and consistent starting point for examining WGS data for prospective laboratory-based surveillance of VTEC O157:H7, but however, the data will continue to be interpreted according to context and in combination with epidemiological and food safety evidence to inform public-health decision making in Canada.

**Electronic supplementary material:**

The online version of this article (10.1186/s12864-018-5243-3) contains supplementary material, which is available to authorized users.

## Background

To protect and promote the health of Canadians, rapid and accurate identification of foodborne pathogens is of paramount importance for effective laboratory-based surveillance and outbreak detection, and is dependent on well-established, standardized and highly discriminatory typing methods [1–3]. For nearly two decades, pulsed-field gel electrophoresis (PFGE) has been the laboratory gold standard for foodborne bacterial subtyping and has been the primary method used by PulseNet Canada, Canada’s national molecular subtyping network for foodborne bacterial disease cluster detection and outbreak response [4, 5]. The most notable strength of applying PFGE not only lies in the expertise and experience in interpreting the data, but also, in the amount of historical PFGE data that exists in Canada’s national databases [4, 6, 7]. Together, this has allowed for meaningful comparisons of contemporary isolates relative to baseline data and in turn, increases the likelihood of identifying significant matches across multiple jurisdictions, which can be further assessed by epidemiologists at the Public Health Agency of Canada’s Outbreak Management Division for potential outbreak response [7]. Despite the historical usefulness and robustness of PFGE, it has been widely demonstrated by the scientific community that the method does not consistently provide optimal discrimination, particularly for highly clonal strains, resulting in outbreak investigations of seemingly unrelated cases and in some instances, discriminates among epidemiological related isolates, obscuring useful linkages and cluster detection [3, 8, 9]. To enhance resolution, multilocus variable-number tandem-repeat analysis (MLVA) was implemented (in Canada, as a supplemental subtyping tool, and elsewhere as a primary tool), and has been extremely valuable in discriminating among closely related isolates that would otherwise be indistinguishable by PFGE [4, 10–15]; however, optimal resolution is often achieved when the results of these typing methods are combined and interpreted together [5], rendering characterization of foodborne bacterial pathogens to be laborious, time-consuming and expensive, which has prohibited widespread adoption of both methods in many public health laboratories [16, 17]. Additionally, MLVA protocols have only been developed for selected pathogens (i.e., *Salmonella* Enteritidis, *Salmonella* Typhimurium and *Escherichia coli* O157:H7) within the PulseNet Canada network, further hindering its overall effectiveness [17]. Despite the utility of MLVA, isolates demonstrating related MLVA profiles may not always be epidemiologically related [18].

With the recent advancements in next generation sequencing (NGS) technologies, and decreasing financial costs, whole-genome sequencing (WGS), which provides the complete genetic blueprint of an organism, has become an increasingly popular method for use in public health laboratories. Since the entire genome is readily available for interrogation, WGS has the capacity to fully replace other preexisting conventional methods for characterizations including serotype, virulence and antimicrobial resistance as these clinically important phenotypes can be predicted (in silico) from the genotype with relative ease [19–21]. In recent years, WGS-based analyses have been proven instrumental in facilitating both the detection and investigation of outbreaks and are rapidly becoming more prominent in public health laboratories as the primary subtyping tool of choice for the characterization of foodborne pathogens (mainly *Listeria monocytogenes* and/or *Salmonella* species) and to support epidemiological investigations [22–25]. The well-recognized capacity of WGS to revolutionize global foodborne disease surveillance has positioned this tool as the prime candidate for replacing molecular subtyping within the PulseNet International network, a global laboratory network comprising 86 countries dedicated to bacterial foodborne disease surveillance [26]. As an integral part of the PulseNet International network, PulseNet Canada has made immense strides towards routine implementation of WGS for foodborne pathogen surveillance; however, despite these efforts, the authors recognize this work is largely underrepresented by Canadian institutions and researchers with limited studies published over the past few years [27–30]. The shortage of Canadian studies documenting the use of WGS for foodborne bacterial pathogen surveillance serves as the primary basis for this study.

In accordance with the existing literature, the authors note that a majority of WGS studies largely report on the use of this method to retrospectively characterize previously identified outbreaks and/or to compare WGS derived typing data with traditional typing methods, such as PFGE and/ or MLVA [31]. Although plentiful, these validation studies are critical for verifying the robustness and technical performance of WGS in comparison to current gold standard methods, especially since WGS is poised to become the new gold standard of bacterial typing [26]. As stated in the PulseNet International Vision paper “any modifications on existing methods or introduction of new methods must be carefully validated and implemented by *all* network members in order to be effective and to avoid disrupting the surveillance due to backwards incompatibility issues” [26]. Hence, this work will contribute to the validation of WGS for foodborne bacterial pathogen surveillance from the Canadian perspective and further strengthen the foundation upon which experts can continue to build upon for implementing and standardizing WGS on a global scale, the primary goal of PulseNet International [26].

In this study, we verify the performance of WGS as superior to current gold standard molecular subtyping methods (i.e., PFGE/MLVA) used by PulseNet Canada for eight well-characterized, retrospective outbreaks of Verotoxigenic *Escherichia coli* (VTEC) O157:H7, which is one of the top pathogens causing the greatest number of hospitalizations (246) and deaths (8) attributed to domestically acquired foodborne illness in Canada each year [32]. VTEC O157:H7 infections, commonly transmitted via the fecal-oral route following ingestion of contaminated foods and/or water, can present a diverse spectrum of clinical manifestations including watery diarrhea, hemorrhagic colitis (HC) and in the most extreme cases, hemolytic uremic syndrome (HUS), which is often fatal for young children, the elderly and immunocompromised individuals [33]. In light of the potential severity of these infections, timely response to potential outbreaks of VTEC O157:H7 is critical, making this pathogen a prime candidate for this study.

Since real-time implementation and routine use of WGS in public health laboratories is highly reliant on standardized data analysis tools [22, 26], we  also provide a comparative analysis of two popular methodologies for WGS analyses, including a single nucleotide variant (SNV) based approach (i.e., the in-house developed single nucleotide variant phylogenomics (SNVPhyl) pipeline) [34] and a wgMLST approach (i.e., the whole genome multilocus sequence type (wgMLST) schema for *Escherichia coli-Shigella* (BioNumerics, Applied Maths, Belgium)) [35]. In brief, SNV-based methods are based on the detection of single nucleotide changes to infer phylogenetic relatedness using a referenced-based mapping approach [22], while wgMLST, an extension of traditional multi-locus sequence typing (MLST), is a gene-by-gene comparative approach that detects allelic variation within a set of microbial genomes using a predefined set of pan-genomic loci [26]. To the knowledge of the authors, this is the first time a SNV-based method has been directly compared to a wgMLST approach for VTEC O157:H7 outbreak detection.

To further strengthen the findings of the current study, we also aimed to describe the number of allele or SNV differences observed among outbreak-related isolates using epidemiological information collected through outbreak investigations and the Public Health Agency of Canada’s Outbreak Management Division’s WGS validation study. This information will provide a useful and consistent starting point for examining laboratory-based surveillance data for VTEC O157:H7, which can be used to inform public-health decision making in Canada.

## Results

### Comparison of WGS with gold standard methods PFGE and MLVA

For the eight outbreaks investigated in this study, very few genetic differences were observed; outbreak-related isolates differed by less than 10 SNVs/ wgMLST alleles, while non-outbreak isolates (i.e., sporadic) differed from the closest neighboring outbreak isolate by greater than 16 SNVs/ 18 wgMLST alleles. This intra-outbreak variation served as the baseline for assessing the impact of WGS on the categorization of outbreak-related cases in comparison to PFGE and MLVA. For most of the outbreaks investigated, the categorization of isolates by WGS as outbreak-related or not outbreak-related was mostly concordant with the results previously obtained for traditional typing methods PFGE and MLVA (Table 1) except for Outbreaks 2, 3, 4 and 6. One additional isolate, which was previously excluded from the investigation based on an unrelated MLVA profile, was observed to group with isolates in Outbreak 2 by 7 SNVs/ ≤ 10 wgMLST alleles suggesting it may have been outbreak-related. Similarly, a single isolate grouping by 0–1 SNVs/ 0–8 wgMLST alleles with isolates in Outbreak 4 was identified by WGS and may have been related. Interestingly, this isolate also demonstrated a related MLVA profile. For Outbreak 3, WGS identified two clinical isolates as potential outliers; these isolates differed by > 23 SNVs/ 23 wgMLST alleles from all other isolates included in the outbreak. Four food isolates differing by > 37 SNVs/ 38 wgMLST alleles also appeared to be outliers by WGS for Outbreak 6. Interestingly, a previously characterized non-outbreak isolate was observed to group with one of the food isolate outliers identified in Outbreak 6 by few genetic differences (1 SNV/ 3 wgMLST alleles). Since this isolate had both an unrelated PFGE pattern and MLVA profile, this isolate would not have been previously identified by traditional typing methods.

**Table 1:** Impact of Whole Genome Sequencing on the Categorization of Outbreak-Related Isolates

Outbreak	Number of Cases Identified by PFGE/MLVA	Number of Cases Identified by WGS	Number of Additional Cases Included by WGS Only^c^	Number of Cases Excluded by WGS
1	14	14	0	0
2	2	3	1	0
3	15	13	0	2^b^
4	23	24^a^	0	0
5	18	18	0	0
6	9	6	1	4^b^
7	31	31	0	0
8	28	28	0	0

*PFGE* pulsed-field gel electrophoresis, *MLVA* multilocus variable-number tandem-repeat analysis, *WGS* whole genome sequencing

^a^Isolate ruled in by WGS also demonstrated a related MLVA profile

^b^Isolates were associated with a suspected multi-strain event; may represent genetic variants for which no clinical/non-clinical match was identified

^c^Isolates were not previously identified as related by PFGE and/or MLVA

### Epidemiological concordance and genetic diversity among outbreak-related isolates

The categorization of cases by WGS was highly concordant with previously acquired epidemiological information for almost all outbreaks in this study. Within each outbreak, very few genetic differences (i.e., < 10 SNVs and wgMLST alleles) were observed between epidemiologically related isolates (Table 2), unless the outbreak was suspected to be multi-strain based on epidemiologic information collected during the investigation (i.e., Outbreak 3 and Outbreak 6). Of note, both multi-strain outbreaks were associated with the consumption of contaminated ground beef products.

**Table 2:** Features of Eight Outbreaks of Verotoxigenic *Escherichia coli* O157:H7 Characterized by Whole Genome Sequencing in Canada

						Number of Genetic Differences Among Outbreak-Related Isolates	
Outbreak	Year	Number of Lab Confirmed Cases	Suspect / Confirmed Source	Number of Implicated PFGE Patterns / MLVA Profiles^d^	Number of Isolates Sequenced in the Study	SNVs	wgMLST alleles
1	2011	14	Raw shelled walnuts	1 / 2	14	0–3	0–4
2	2011	2	In-shell hazelnuts	1 / 2	2	0	4
3^a^	2012	15	Preformed beef patties	6 / 8	42^b^	Group 1^c^: 0–1 Group 2^c^: 0–1 Group 3^d^: 0 Group 4: 0–3 Group 5^c^: 0–1 Group 6^d^: 0 All Isolates: 0–110	Group 1^c^: 1–5 Group 2^c^: 0–3 Group 3^d^: 1–4 Group 4: 0–9 Group 5^c^: 1–3 Group 6^d^: 2–4 All Isolates: 0- > 100
4	2012	23	Romaine lettuce	2 / 1	23	0–1	0–7
5	2012	18	Beef products	1 / 1	25^b^	0–3	0–4
6^a^	2012	9	Frozen beef burgers	7 / 8	36^b^	Group 1^d^: 0–2 Group 2^d^: 0 Group 3^d^: 0–1 All Isolates: 0–106	Group 1^d^: 0–4 Group 2^d^: 0–3 Group 3^d^: 0–2 All Isolates: 0- > 90
7	2012	31	Fast Food lettuce	3 / 1	31	0–2	0–8
8	2013	28	Raw milk cheese	4 / 4	44^b^	0–5	0–9

*PFGE* pulsed-field gel electrophoresis, *MLVA* multilocus variable-number tandem-repeat analysis, *SNVs* single nucleotide variants, *wgMLST* whole-genome multilocus sequence typing

^a^Suspected multi-strain event; isolates split into multiple groupings and one or more potential outliers

^b^One or more food isolates were sequenced

^c^Only includes food isolates

^d^Contains clinical and food isolates

Outbreak 3 (2012) included a total of 15 laboratory confirmed cases and 27 food isolates with multiple PFGE pattern combinations and MLVA profiles. All thirteen cases with exposure information reported consuming or probably consuming ground beef, while two cases were lost to follow-up. The food safety investigation identified several ground beef products produced at the same establishment that were contaminated with VTEC O157:H7; five different PFGE pattern combinations were identified through product testing. Approximately 0–110 SNVs/ 0- > 100 wgMLST alleles were detected among all isolates; however, these isolates were observed to split into six distinct groupings and was consistent with previous subtyping methods as each group included isolates with distinct PFGE pattern combinations and MLVA profiles. Among the six groupings, 34 SNVs/ 27 wgMLST alleles or more were detected while isolates within each group differed by ≤3 SNVs/ ≤9 wgMLST alleles. Three of the six groupings included one or more clinical isolates (Group 4) as well as some food isolates (Group 3 and Group 6), while the remaining 3 groups only contained food isolates (Group 1, Group 2 and Group 5). The results obtained from WGS were consistent with epidemiological evidence; clinical cases with common exposures were also found to be closely related by WGS. With respect to the two clinical outliers identified by WGS, both isolates had PFGE pattern combinations and/ or MLVA profiles that were distinct from the other outbreak isolates. Interestingly, exposure to ground beef was reported for one of these cases, while the other case was lost to follow-up.

Similar findings were observed for Outbreak 6 (2012), which included a total of 9 laboratory confirmed cases and 27 food isolates obtained from the food safety investigation. Eight cases reported consuming one of two different brands of frozen beef burgers produced at the same establishment (Brand A and Brand B); epidemiological information was not available for the remaining case. WGS detected a high level of genetic variability among outbreak isolates (0–106 SNVs/ 0- > 90 wgMLST alleles). Three distinct groupings, each containing clinical and food isolates were observed and differed from one another by greater than 78 SNVs/ 80 wgMLST alleles. Within each group, less than 3 SNVs/ 5 wgMLST alleles were detected. Upon further review of epidemiologic information, it was noted that all cases included in Group 1 (*n* = 3) reported exposure to Brand A frozen beef burgers, while all cases in the Group 2 (*n* = 5) reported exposure to Brand B. Consistent with epidemiological findings, Group 1 only included food isolates from Brand A frozen beef burgers, while Group 2 only included food isolates from Brand B. Group 3 also contained food isolates from closed samples of Brand B frozen burgers, however, these isolates had a different production date than those in Group 2. The clinical isolate with no exposure information available was observed to group with two of the Brand B food isolates in Group 3 by very few genetic differences (1 SNVs/ 0 wgMLST alleles) suggesting this clinical case was likely related to the outbreak. Outside the three WGS groupings, there were four food isolates that did not group with any other isolates in Outbreak 6 as they differed by greater than 37 SNVs/ 38 wgMLST alleles from the closest neighboring clinical outbreak isolate. This observation was not unexpected as these food isolates were obtained from frozen beef products with different production dates than those represented in the rest of the cluster and had PFGE pattern combinations and MLVA profiles that were distinct from the other outbreak isolates.

### Inter-outbreak variability

The eight outbreaks were visibly delineated from one another by WGS. On average, outbreaks differed by 18 SNVs/ 24 wgMLST alleles or more except for Outbreak 3 (Group 2) and Outbreak 6 (Group 2), which differed by less than or equal to 13 SNVs/ 18 wgMLST alleles and Outbreak 4 and Outbreak 7, which differed by less than or equal to 10 SNVs/ 16 wgMLST alleles. Of note, no food isolates were available for WGS analyses for Outbreak 4 and Outbreak 7 as the pathogen was not isolated from the implicated food commodity.

### Comparison of SNVPhyl and wgMLST

**Fig. 1:**
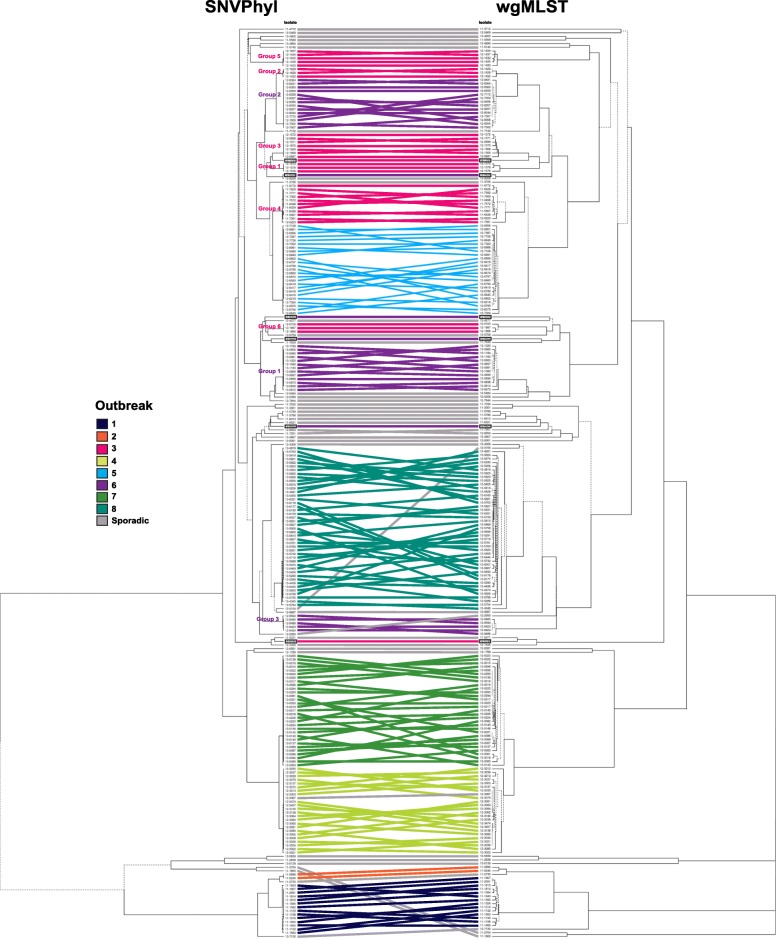
Side by side comparison of SNVPhyl and wgMLST. The tanglegram was constructed in R Studio using the dendextend package and consists of 250 clinical and non-clinical Verotoxigenic *Escherichia coli* O157:H7 isolates from eight multijurisdictional outbreaks and 41 temporally-related non-outbreak isolates. To maximize isolate matching between trees the layout was optimized using the step2side method. Branches that are unique to each tree are indicated with dotted lines. Identical strains are linked between trees using straight lines that are colored according to outbreak. Outliers are indicated with black boxes and connections between sporadic isolates are colored grey

Based on the side by side comparison of SNVPhyl and wgMLST (Fig. 1) both methods were equally successful in distinguishing outbreak-related from non-outbreak related isolates. In Fig. 1 several crossings were observed among connecting isolates within each outbreak between the two trees, however, this was not unexpected. Since SNVPhyl and wgMLST measure isolate relatedness based on different aspects of the genome (i.e., SNVs vs. alleles), the clustering of isolates within each outbreak is not anticipated to be identical; isolates that appear more closely related in one outbreak by wgMLST (i.e., 1 allelic difference) may not be as closely related by SNVPhyl (i.e., 2 or 3 SNV differences) for that same outbreak, and as a result, will appear in different positions on the trees. This observation is likely attributed to the foundational concept of wgMLST, which is based on allelic variation. Unlike SNV-based methods, wgMLST considers recombinations and insertions/ deletions as single evolutionary events; therefore, a single allelic difference can encompass multiple SNVs. Despite these minor differences, the tree topologies generated by SNVPhyl and wgMLST were highly similar. To further strengthen these findings, the Fowlkes-Mallows (FM) Index was calculated to determine the level of similarity between the SNVPhyl and wgMLST trees and then compared to the critical value, which takes into account the relevant Expectancy (E_FM) and Variance (V_FM) values under the null hypothesis of no relation at α = 0.05. Since the FM Index was higher (0.987) than the critical value (0.854), the conclusion that SNVPhyl and wgMLST lead to similar topologies is statistically supported.

## Discussion

As demonstrated in this study, WGS provides enhanced resolution over traditional subtyping methods and accurately distinguishes outbreak-related isolates from non-outbreak related isolates with high epidemiological concordance. WGS has the capacity to identify isolates demonstrating variant PFGE patterns/MLVA profiles that would otherwise be overlooked with traditional subtyping methods for cluster detection, and as a result, impacted the categorization of cases for 50% of the outbreaks investigated in this study. Despite the prior exclusion of a single isolate from Outbreak 2 based on MLVA, the relatively few genetic differences (7 SNVs/ ≤10 wgMLST alleles) detected by WGS could suggest this isolate was actually related, but however, inadvertently ruled-out due to the lack of resolution provided by MLVA. Similarly, WGS proved useful in identifying a non-outbreak isolate that grouped tightly with Outbreak 4 (0–1 SNVs/ 0–8 wgMLST alleles), however, this isolate was observed to have a related MLVA profile (based on PulseNet Canada standardized interpretation guidelines) to that of the other isolates included in this outbreak. Had MLVA been routinely used to characterize all VTEC O157:H7 in Canada, this isolate would have been detected prior to the WGS era. At the time of the investigation, MLVA was primarily used to provide enhanced resolution to selected outbreaks but was not officially implemented for routine characterization of VTEC O157:H7 in Canada until 2013. Historically, given the overall effectiveness of MLVA for VTEC O157:H7 [4, 5], the impacts of routine sequencing may not be as substantial for surveillance and cluster detection of this pathogen in Canada; other priority pathogens such as *Listeria* and *Salmonella*, for which no interim method offering comparable resolution is available, have taken precedence for WGS implementation in a number of different countries including Denmark, England and the United States [31]. PulseNet Canada has followed in similar footsteps; routine sequencing of all *Listeria* and *Salmonella* species was implemented in Canada in January and May of 2017, respectively.

From an operational standpoint, WGS has already proven useful for categorizing isolates as related/ unrelated despite indistinguishability by MLVA; a recent multijurisdictional cluster of VTEC O157:H7 demonstrating highly related MLVA profiles truly turned out to be a single-provincial cluster after a number of outliers were identified by WGS, which was also supported by epidemiological evidence (data not shown). The resources needed to investigate multijurisdictional outbreaks are substantial; these highly limited resources would have been much more effectively allocated if WGS had been used at that time instead of molecular methods. Therefore, it is clear that the use of traditional subtyping methods such as PFGE and/or MLVA to define an outbreak-related case can result in misclassification, and in turn, impede cluster detection and epidemiologic investigations as isolates demonstrating indistinguishable or highly similar subtyping results can arise from different sources, while isolates deemed unrelated by traditional typing methods could have originated from the same source as reported in this study and elsewhere [8, 9, 18].

Routine and real-time implementation of WGS has the potential to detect and better support successful investigations by reducing the possibility of false implication by lower resolution methods (i.e., PFGE and MLVA) and more appropriately direct epidemiological resources to investigating cases that are more likely to actually be linked. This will be critical for making further strides in reducing the morbidity and mortality associated with this pathogen [23]. More importantly, WGS will lead to a better understanding of so-called sporadic cases of illness as demonstrated in this study; a previously characterized non-outbreak clinical isolate with a variant PFGE pattern and unrelated MLVA profile was observed to group with one of the food isolate outliers from Outbreak 6 by few genetic differences (1 SNV/ 3 wgMLST alleles). Interestingly, both isolates were collected within the same time frame and in the same province. Therefore, it is quite possible this “sporadic” clinical case was not sporadic at all, but rather outbreak-related and was overlooked with traditional subtyping methods applied at that time. From these results, it is apparent that WGS has the ability to link sporadic cases of illness to potential food sources at a level of confidence that could not previously be achieved with traditional subtyping methods. More recently, WGS elucidated a possible linkage between a single case of *Listeriosis* in the United States and a cross-border recalled lettuce product, which was also identified as a novel vehicle for *Listeria* at the time of the investigation [36]. The increased resolution provided by WGS for cluster detection and outbreak support is clearly demonstrated in this study, however, since epidemiological information was not available for the non-outbreak isolates described above, little can be said about whether they were truly outbreak-related, which was a major limitation of this study.

In addition to providing enhanced resolution over traditional typing methods, WGS can also provide additional resolution for multi-strain outbreak investigations. Two outbreaks examined in the study, Outbreak 3 and Outbreak 6, consisted of both clinical and food isolates possessing multiple PFGE patterns and MLVA profiles. Since a number of variant PFGE patterns and MLVA profiles were collected from the food products, it was suspected that each outbreak was associated with a multi-strain event. This hypothesis was confirmed by WGS; isolates within each outbreak split into multiple, distinct groups differing by more genetic differences (34 SNVs/ 27 wgMLST alleles or more) than what was identified among isolates contained within each of those groups (≤5 SNVs and ≤ 9 wgMLST alleles) and when compared to other outbreaks studied herein. As well, multiple isolates were identified for both Outbreak 3 (*n* = 2 clinical isolates) and Outbreak 6 (*n* = 4 food isolates) that did not group with any isolates in the respective outbreak based on WGS, which could suggest these isolates were unrelated. However, given these outbreaks were multi-strain events, it is possible these genetic outliers were outbreak-related, but were not genetically related to other confirmed cases/ isolates. With respect to the food isolate outliers, several scenarios are plausible. It is possible that: clinical illness did not result from that particular strain; that illness did occur, but was not captured due to the under-reporting of enteric illness in Canada; the case(s) bore a PFGE pattern different from that of the food isolate, and therefore, was excluded; or that the product sampling and testing that was completed as part of the food safety investigation went beyond the scope of the implicated food product. The final scenario is not uncommon during food safety investigations as a variety of inputs and lot codes are often tested to determine the scope of the contamination. A similar justification can be made for clinical outliers. To mitigate this issue, analysis of all sporadic isolates collected during an outbreak time frame would be required to achieve the highest level of resolution [17]. Due to lack of resources, not all sporadic isolates collected during the time frame of the outbreaks examined could be sequenced and was another limitation of the study. This illuminates the importance of characterizing all cases in real-time as part of routine laboratory-based surveillance (versus a subset of representative cases). Although laboratory evidence is critical to identify clusters, and to link or exclude cases, it remains limited in its capability to identify multi-strain events. This aspect of laboratory surveillance remains unchanged in the WGS era – only epidemiological and food safety evidence analyzed in an integrated manner will detect and confirm outbreaks involving more than one strain.

The results of this study also demonstrate the potential of WGS to identify related cases of illness spanning multiple years as well as illuminate possible transmission pathways that could not previously be explored with tradition typing methods. In comparison to the other outbreaks examined in this study, a portion of isolates from Outbreak 3 (Group 2) and Outbreak 6 (Group 2) grouped much closer to each other (≤13 SNVs/ ≤18 wgMLST alleles), as well as isolates in Outbreak 4 and Outbreak 7 (≤10 SNVs/ ≤16 wgMLST alleles). Although Outbreak 3 and Outbreak 6 were distinct events in time and space (i.e., Outbreak 3 occurred between September 2011 and March 2012, while Outbreak 6 took place between October 2012 and March 2013), the genetic similarity between the isolates involved, and commonality between implicated products (i.e., ground beef products), could suggest a shared upstream origin of contamination. Given the complexity and potential multitude of inputs that can be incorporated during the production of ground beef products, WGS may present avenues to help uncover potential linkages between production facilities, the food processing environment, and upstream sources of contamination. Although the mechanism of contamination for Outbreak 4 and Outbreak 7 was unknown, the lower level of genetic diversity between the two events, in addition to the presence of related MLVA profiles could suggest these outbreaks, which occurred approximately one year apart, also had some common upstream source of contamination (i.e., on the farm, in processing, etc.). Alternatively, it is also possible these outbreaks were distinct and unrelated events, but however, involved a prevalent strain of VTEC O157:H7 that has evolved over time, which would account for the moderate level of genetic variation observed between the two outbreaks. The detection of potentially related clusters of isolates from multiple years by WGS raises the question of whether narrow and predefined search windows (i.e., illness onset observed within the same 60-day period of time) will still be appropriate for WGS-based analyses, and forces consideration of the broader impacts on laboratory-based surveillance with respect to how clusters are identified. While the narrow search window is relevant to detection of isolated point source outbreaks, WGS provides an opportunity to connect seemingly independent events in space and time. The ability to link clusters and events over time has already proven useful for hypothesis generation within the PulseNet Canada network; clusters that are more closely related by WGS are more likely to be associated with similar sources/exposures than those that are more distantly related. In the present study, this was illustrated by the closer distances seen between Outbreaks 3 and 6 (linked to ground beef) and Outbreaks 4 and 7 (linked to lettuce). More recently, it has become routine practice in Canada to check for “historical matches” when multijurisdictional clusters of interest are identified.

Even with the advancements in technology and costs, and undeniable advantages of WGS over traditional typing methods, many obstacles and limitations still exist with respect to implementation, particularly on a global scale. Presently, the most challenging hurdle to overcome is the harmonization of a single analysis method. Since WGS has taken center stage, there has been no shortage in the development of open-source bioinformatics tools, SNV-pipelines or commercially available software for analyzing WGS data in the context of foodborne pathogen surveillance and cluster detection [22, 26, 37]. In this study, two popular analysis methods were assessed, including a SNV-based approach (i.e., SNVPhyl) and wgMLST (the analysis method of choice for PulseNet International), to assess their utility and comparability for outbreak detection using eight well-characterized retrospective outbreaks of VTEC O157:H7 in Canada. As expected, the results generated by both SNVPhyl and wgMLST were highly comparable with strong statistical support indicating the methods led to similar topologies (FM Index = 0.987). Although the grouping of outbreak-related isolates was virtually the same between analysis methods, there were noticeably fewer SNV differences than there were allele differences. Again, this was not unanticipated as the methods evaluate different aspects of the genome. Since wgMLST evaluates allelic variation among pan-genomic loci, more genetic differences are likely to be detected as more genetic sites are considered. In contrast, SNVPhyl examines single nucleotide changes associated with the shared genome (i.e., SNVs that are common to both the reference and the collection of microbial genomes under analysis), and therefore, the number of genetic sites available for comparison is highly dependent on the reference selected and the overall genetic diversity of the dataset. The dependency on appropriate reference selection to identify SNVs may be viewed as a disadvantage of SNV-based approaches; different references could potentially generate different SNV profiles, and as a result, impact inter-laboratory comparisons [38]. Even though the SNVPhyl results demonstrated high epidemiological concordance, and the number of SNVs detected among outbreak-related isolates in this study was comparable to those previously reported for VTEC O157 (i.e., ≤ 5 SNVs) [8, 9, 27, 39–41], the significant technical limitations surrounding this methodology, as previously discussed in [26], does not make this approach suitable for global implementation, but however, could be useful for providing additional resolution to outbreak investigations if required.

Unlike SNV-based approaches, wgMLST satisfies many critical needs required by public health laboratories to deliver accurate and real-time surveillance of foodborne pathogens. Not only does the method provide sufficient resolution and epidemiological concordance as indicated in this study, but also, it is easily scalable and amenable to both standardization and stable nomenclature [26]. In light of these technical advantages, wgMLST has been selected by PulseNet International as the analysis method of choice in the world’s first attempt at standardizing a single genomic method for truly global foodborne disease surveillance [26]. To the knowledge of the authors, the use of wgMLST for surveillance and cluster detection has not yet been documented for VTEC O157:H7 in the scientific literature, however, the technique has been found useful for other foodborne pathogens including *L. monocytogenes* [37, 42–44], *Campylobacter spp.* [45], and *S.* Enteritidis [38, 46]. In a recent publication by Holmes et al., it was demonstrated that the core genome multilocus sequencing typing approach (cgMLST) in BioNumerics v7.6 generated comparable resolution to that of the Public Health England single nucleotide polymorphism (SNP) semi-automated bioinformatics pipeline; related isolates were clearly distinguished from unrelated isolates for VTEC O157 [47]. Despite the effectiveness of cgMLST, this method is slightly less discriminatory than SNP-based methods as the number of core loci differing between unrelated isolates is often much lower than the number of SNPs detected between those same isolates [47]. Based on more recent experiences at PulseNet Canada, this observation was also noted during a large-scale retrospective laboratory validation project to develop and evaluate WGS as the primary laboratory tool for foodborne bacterial disease surveillance and cluster detection (unpublished data). Although a slightly lower level of resolution is not unexpected as a smaller proportion of the genome is examined for cgMLST opposed to SNV-based approaches, the potential for isolates to appear more related than they actually are would suggest cgMLST is not optimal for continuous monitoring activities such as surveillance and/or cluster detection. In spite of this limitation, the inherent stability and reproducibility of cgMLST, which is based on a conserved set of well-defined species-specific loci readily available in the public domain [48], has given this analysis method the upper hand over wgMLST. The inclusion of pseudogenes and paralogous genes within the present wgMLST schema, which could potentially lead to inaccurate clustering and misleading relationships among isolates of interest, has raised some concerns within the scientific community [37, 46, 47]. To address these concerns, global initiatives are currently ongoing to standardize wgMLST allele databases for a number of priority foodborne pathogens, which will be developed and curated by subject-matter experts from both public health laboratories and academia to ensure the most appropriate reference alleles are selected in order to minimize the presence of highly variable genetic elements that could potentially skew true genetic relationships [26, 49].

Based on the findings of the present study and experience investigating a wider variety of clusters and outbreaks in Canada, VTEC O157:H7 isolates associated with the same outbreak had 10 or fewer wgMLST allelic differences (< 10 SNVs), whereas isolates from different epidemiological events were more than 15 wgMLST alleles different. While establishing “cutoffs” or threshold values to define the maximum number of allele/SNV differences needed to define isolates as “matching” may be an attractive idea, determining whether the level of diversity within a group of isolates indicates a common source of illness is extremely challenging in reality. Since diversity (i.e., range of pairwise genetic distances) is expected to increase with the effective population size, it is highly unlikely that a single distance threshold will be able to consistently predict whether isolates will be epidemiologically related or not [43, 47]. Multiple streams of evidence must be taken into consideration when evaluating the level of relatedness among isolates using WGS data. This is reminiscent to the interpretation of PFGE results to define “matches”, which evolved from the use of Tenover’s criteria (i.e., number of bands different) to organism-specific guidelines highly informed by the context of the isolates in question and historical baselines [50–52]. To avoid limiting the richness and continuous nature of WGS data, the values reported in this study should not serve as threshold cutoffs; rather, they provide a useful and consistent starting point for examining laboratory-based surveillance data for the purposes of public health decision-making. Ultimately, a standardized strain nomenclature could be used to automate cluster detection, more specifically, as the initial step in the cluster detection process to identify closely-related isolates. This would essentially eliminate the need to explain the topology of a phylogenetic tree, thereby allowing for more rapid communication and sharing of results among stakeholders for the purposes of detecting common source outbreaks, upon which could be further investigated using higher resolution methods like wgMLST [26, 49]. Such a system would truly enable global foodborne disease surveillance.

## Conclusions

This retrospective study serves as proof of principle and clearly demonstrates increased genetic resolution of WGS for cluster detection and outbreak support of VTEC O157:H7 in Canada over traditional subtyping methods (i.e., PFGE and MLVA). The use of WGS in routine surveillance will not only enhance cluster detection but also provide guidance and support for more targeted epidemiological investigations. Following this successful validation WGS is now routinely applied to all *E. coli* isolates submitted through the PulseNet Canada network.

The high level of congruency with respect to the topologies generated by SNVPhyl and wgMLST indicates either one of these methods would be appropriate to use for WGS-based analyses of VTEC O157:H7 as they were both equally successful in distinguishing outbreak-related isolates from non-outbreak-related isolates. Given the technical advantages of wgMLST, this methodology is more suitable for facilitating truly global foodborne disease surveillance. However, until a global allele database has been established, this study validates the current BioNumerics v7.6 wgMLST schema based on 17,380 loci as a suitable version for surveillance and cluster detection of VTEC O157:H7 in the interim, and has been implemented as the primary tool for surveillance and cluster detection of VTEC O157:H7 within the PulseNet Canada network. Although not routinely applied, SNVPhyl may be used in situations where additional resolution is necessary. As it was not assessed in this study, additional work will have to be conducted to determine the effectiveness of this schema for other *E. coli* serotypes.

As with the implementation of any new method, cost considerations must be taken into account and will be highly dependent on the resources and capabilities of the laboratory itself. To truly benefit from the robustness of wgMLST, the allele database will need to be software-naïve and hosted on a platform that is openly accessible to ensure standardization across different organizations and encourage widespread adoption. Lastly, to avoid limiting the richness and continuous nature of WGS data, the number of allele (or SNV) differences observed among outbreak-related isolates in this study are not intended to be used as stringent cut-offs. These values will provide a useful and consistent starting point for examining laboratory-based surveillance data for VTEC O157:H7, but however, cluster detection and response will continue to be interpreted according to the context, and in combination with epidemiological and food safety evidence to inform public-health decision making in Canada.

## Methods

### Isolate selection

A total of 250 clinical and non-clinical VTEC O157:H7 isolates consisting of 141 laboratory-confirmed clinical cases and 68 food isolates from 8 multijurisdictional foodborne outbreaks and 41 temporally-related non-outbreak isolates (*n* = 40 clinical; *n* = 1 environmental) were selected for the study. An outbreak was defined as a National investigation led by the Public Health Agency of Canada’s Outbreak Management Division. The 8 outbreaks analyzed in this study were selected from 14 multijurisdictional outbreaks identified between 2011 to 2013 based on the following inclusion criteria: the outbreak was “solved” (i.e., a suspect or confirmed source was identified using conventional methods); the outbreak strains spanned the diversity of subtypes (PFGE/MLVA) typically seen in Canada during the study time period (2011–2013); the outbreak had cases in more than one province or territory, and all laboratory-confirmed human-clinical cases included in the outbreak had sequence data available. Forty-one non-outbreak related isolates occurring within 60 days of the selected outbreaks were included in the study based on molecular subtype (PFGE and/or MLVA). The number of days was measured according to the date the isolate was uploaded to the national PulseNet Canada PFGE database.

### Outbreak epidemiological information

Preliminary epidemiological information was collected from cases with *E. coli* O157:H7 infection during routine public health follow up. Where possible, cases were re-interviewed with outbreak specific questionnaires and loyalty card information/purchase records obtained in order to validate reported exposures. All data was analyzed centrally by the Public Health Agency of Canada’s Outbreak Management Division.

### Molecular subtyping

Isolates were characterized by PFGE by provincial public health laboratories, the Canadian Food Inspection Agency (CFIA), and the National Microbiology Laboratory (NML) following standardized PulseNet International protocols [53]. MLVA was performed as per Rumore et al at the NML [5]. Centralized analysis of PFGE results was performed following published criteria [52] and relatedness of MLVA profiles was measured using PulseNet Canada standardized interpretation guidelines: isolates differing by no more than one repeat at ≤3 loci or up to 3 repeats at a single locus and no variation at VNTR 34 (i.e., the second integer in the MLVA profile), are considered related [5]. Molecular subtyping results were generated in real-time as part of routine laboratory-based surveillance and were not repeated for this study.

### Sequencing

Whole-genome sequencing was primarily performed at the National Microbiology Laboratory in Winnipeg; some sequencing was also performed at the CFIA. Genomic DNA was obtained from a single colony incubated at 37 °C overnight on nutrient agar. DNA was extracted using the metagenomic DNA isolation kit for water (Epicentre, now Illumina, San Diego, CA) or the DNeasy blood and tissue kit (Qiagen, Valencia, CA). Libraries were prepared using the Nextera XT DNA library prep kit (Illumina) according to the manufacturer’s guidelines. Sequencing was performed on the Illumina MiSeq platform with the MiSeq Reagent Kit V3 (Illumina) to achieve average genome coverage of greater than or equal to 50x for all isolates. Raw sequence data was deposited into the Integrated Rapid Infectious Disease Analysis (IRIDA) platform (www.irida.ca) hosted at the NML for further downstream analyses.

### Quality control of Illumina MiSeq data

The quality of the raw sequence reads was evaluated using FastQC, Kraken, and BioNumerics (BN) v7.6. As a primary screen, FastQC [54] was used to determine the average genome coverage, of which has been incorporated as an automated pipeline within the IRIDA platform. For reads to be considered good quality, the estimated genome coverage must have been ≥40x. Sequence data demonstrating acceptable coverage was then assessed using Kraken [55]. Greater than or equal to 45% of the reads must have mapped to *Escherichia coli* for the isolate to be submitted for the final stage in the quality assessment. To ensure all potentially problematic isolates were identified and removed prior to analyses, the final quality assessment was conducted using the quality control parameters incorporated into the BN v7.6 platform: a core percent of ≥90% (i.e., at least 90% of the 2, 513 loci contained in the BioNumerics v7.6 core loci schema, which is synchronized with the Enterobase schema [48], must have been identified) and a genome size between 5.0 and 5.6 M base (Mb) pairs were required. Isolates whose quality parameters did not meet these standards were re-sequenced.

### WGS-based subtyping

Sequence analysis was performed using two different methods: 1) in-house developed bioinformatics SNVPhyl pipeline v1.0 for Paired-End data [34] and 2) wgMLST [35]. The following parameters were applied for the SNVPhyl pipeline: mapping coverage of 80%, read coverage of 10x for identification of variants, mean mapping quality of 30 for inclusion of a variant call, SNV abundance ratio of 0.75, 90% identity for a repeat region with a minimum length of 150 base pairs, and a search window of 20 base pairs for high-density SNV regions with a threshold of 2 SNVs to flag high-density SNV regions. This pipeline has also been incorporated into the IRIDA platform. The publically available closed, finished genome Sakai (NC_002695) was used as the reference for this study and the maximum-likelihood phylogenetic tree was constructed using PhyML. The wgMLST schema used in this study was based on the EnteroBase schema [48] with key modifications; the core genome MLST schema was extended to a pan-genomic schema using 289 publically available reference genomes in order to capture the known diversity of *E. coli*. Currently, the schema consists of 17,380 loci consisting of 14,837 accessory loci, 2513 core loci, as well as loci from three smaller traditional MLST schemas containing 7, 8, and 15 MLST loci as defined by Achtman, Pasteur, and Whittam, respectively [35]. For each isolate, locus presence was analyzed, and if present, the allele variant was determined. Any sequence differences detected at a single locus resulted in an allelic difference. If the sequence was determined to be different from the known alleles identified for a particular locus, it was considered to be a new allele and was assigned a unique allele number. Within BioNumerics v7.6, two algorithms are used for identification of alleles, assembly-free and assembly-based. The assembly-free methodology identifies alleles based on the raw sequence reads using a k-mer based approach whereas the assembly-based method identifies alleles based on the de novo SPAdes assembled genomes using BLAST. In order for an allele to be considered “good quality”, the corresponding locus or gene must have been identified by both allele-calling algorithms. A dendrogram was constructed in BioNumerics v7.6 using the categorical (values) similarity coefficient and the unweighted pair group method with arithmetic mean (UPGMA) for hierarchical clustering.

### Figure generation and statistical analysis

To provide a side by side visual comparison of SNVPhyl and wgMLST, distance matrices obtained from both analysis methods were used to generate UPGMA (Unweighted Pair Group Method with Arithmetic Means) dendrograms using the hclust function in R Studio. Using the dendextend package, a tanglegram was generated and layout was optimized using the step2side method. To provide statistical evidence to support the similarity in clustering between SNVPhyl and wgMLST the dendextend package was also used to calculate the Fowlkes-Mallows Index (FM), which measures the similarity in clustering between two methods (i.e., a value closer to 1 indicates a high level of similarity between clusterings), and the Expected FM Index (E_FM) and Variance FM (V_FM) [56]. The E_FM and V_FM indexes correspond to the expectancy and variance values under the null hypothesis (H_0_) at α = 0.05, which implies there is no correlation between the topologies of the SNVPhyl tree and wgMLST tree as one is simply a random shuffle of the other. To reject the null hypothesis at α = 0.05, the critical value E_FM + 1.645*(√ V_FM) must be less than the observed FM.

## Additional file

Additional file 1: BioProject and BioSample information for the 250 isolates used in the study. (DOCX 33 kb)

## Abbreviations

CFIA: Canadian Food Inspection Agency
CLSN: Canadian Laboratory Surveillance Network
CNPHI: Canadian Network for Public Health Intelligence
HC: Hemorrhagic colitis
HUS: Hemolytic Uremic Syndrome
IRIDA: Integrated Rapid Infectious Disease Analysis Platform
MLST: Multilocus sequence typing
MLVA: Multilocus variable-number tandem-repeat analysis
NGS: Next generation sequencing
NML: National Microbiology Laboratory
OMD: Outbreak Management Division
PFGE: Pulsed-field gel electrophoresis
PHAC: Public Health Agency of Canada
SNV: Single nucleotide variant
SNVPhyl: Single nucleotide variant phylogenomics pipeline
UPGMA: Unweighted pair group method with arithmetic mean
VTEC: Verotoxigenic *Escherichia coli*
wgMLST: Whole genome multilocus sequence typing
WGS: Whole genome sequencing
