# Supplementary material for: Evaluation of whole-genome sequencing for outbreak detection of Verotoxigenic Escherichia coli O157:H7 from the Canadian perspective
Source: BMC Genomics. 2018 Dec 4;19:870. doi: 10.1186/s12864-018-5243-3 (PMC6278084; doi:10.1186/s12864-018-5243-3)
Supplement: Supplementary file 1 — BioProject and BioSample information for the 250 isolates used in the study. (DOCX 33 kb) [file 12864_2018_5243_MOESM1_ESM.docx]

**Additional file 1. BioProject and BioSample information for the 250 isolates used in the study.**

| **Sample Name** | **Source** | **BioProject** | **BioSample** | **Sporadic/ Outbreak** |
| --- | --- | --- | --- | --- |
| 11-2735 | Human | PRJNA481261 | SAMN09738481 | Excluded from Outbreak 2 based on MLVA |
| 11-1024 | Human | PRJNA481261 | SAMN09738465 | Outbreak 1 |
| 11-1132 | Human | PRJNA481261 | SAMN09738466 | Outbreak 1 |
| 11-1133 | Human | PRJNA481261 | SAMN09738467 | Outbreak 1 |
| 11-1198 | Human | PRJNA481261 | SAMN09738468 | Outbreak 1 |
| 11-1455 | Human | PRJNA481261 | SAMN09738469 | Outbreak 1 |
| 11-1516 | Human | PRJNA481261 | SAMN09738470 | Outbreak 1 |
| 11-1582 | Human | PRJNA481261 | SAMN09738471 | Outbreak 1 |
| 11-1583 | Human | PRJNA481261 | SAMN09738472 | Outbreak 1 |
| 11-1584 | Human | PRJNA481261 | SAMN09738473 | Outbreak 1 |
| 11-1814 | Human | PRJNA481261 | SAMN09738474 | Outbreak 1 |
| 11-1815 | Human | PRJNA481261 | SAMN09738475 | Outbreak 1 |
| 11-1867 | Human | PRJNA481261 | SAMN09738477 | Outbreak 1 |
| 11-1943 | Human | PRJNA481261 | SAMN09738478 | Outbreak 1 |
| 11-2591 | Human | PRJNA481261 | SAMN09738479 | Outbreak 1 |
| 11-0240 | Human | PRJNA481261 | SAMN09738463 | Outbreak 2 |
| 11-0896 | Human | PRJNA481261 | SAMN09738464 | Outbreak 2 |
| 11-5567 | Human | PRJNA481261 | SAMN09738488 | Outbreak 3 |
| 11-6436 | Human | PRJNA481261 | SAMN09738493 | Outbreak 3 |
| 11-6498 | Human | PRJNA481261 | SAMN09738494 | Outbreak 3 |
| 11-6529 | Human | PRJNA481261 | SAMN09738495 | Outbreak 3 |
| 11-6772 | Human | PRJNA481261 | SAMN09738496 | Outbreak 3 |
| 11-7391 | Human | PRJNA481261 | SAMN09738501 | Outbreak 3 |
| 11-7392 | Human | PRJNA481261 | SAMN09738502 | Outbreak 3 |
| 11-7572 | Human | PRJNA481261 | SAMN09738503 | Outbreak 3 |
| 11-7659 | Human | PRJNA481261 | SAMN09738505 | Outbreak 3 |
| 11-7771 | Human | PRJNA481261 | SAMN09738506 | Outbreak 3 |
| 12-0103 | Human | PRJNA481261 | SAMN09738507 | Outbreak 3 |
| 12-0223 | Human | PRJNA481261 | SAMN09738509 | Outbreak 3 |
| 12-0745 | Human | PRJNA481261 | SAMN09738511 | Outbreak 3 |
| 12-0897 | Human | PRJNA481261 | SAMN09738512 | Outbreak 3 |
| 12-0898 | Food | PRJNA481261 | SAMN09738449 | Outbreak 3 |
| 12-1432 | Food | PRJNA454819 | SAMN09668376 | Outbreak 3 |
| 12-1433 | Food | PRJNA454819 | SAMN09668377 | Outbreak 3 |
| 12-1434 | Food | PRJNA454819 | SAMN09668378 | Outbreak 3 |
| 12-1435 | Food | PRJNA454819 | SAMN09668379 | Outbreak 3 |
| 12-1568 | Food | PRJNA454819 | SAMN09668380 | Outbreak 3 |
| 12-1569 | Food | PRJNA454819 | SAMN09668381 | Outbreak 3 |
| 12-1570 | Food | PRJNA454819 | SAMN09668382 | Outbreak 3 |
| 12-1571 | Food | PRJNA454819 | SAMN09668383 | Outbreak 3 |
| 12-1572 | Food | PRJNA454819 | SAMN09668384 | Outbreak 3 |
| 12-1573 | Food | PRJNA454819 | SAMN09668385 | Outbreak 3 |
| 12-1576 | Food | PRJNA454819 | SAMN09668386 | Outbreak 3 |
| 12-1578 | Food | PRJNA454819 | SAMN09668387 | Outbreak 3 |
| 12-1628 | Food | PRJNA454819 | SAMN09668388 | Outbreak 3 |
| 12-1629 | Food | PRJNA454819 | SAMN09668389 | Outbreak 3 |
| 12-1632 | Food | PRJNA454819 | SAMN09668390 | Outbreak 3 |
| 12-1637 | Food | PRJNA454819 | SAMN09668391 | Outbreak 3 |
| 12-1791 | Human | PRJNA481261 | SAMN09738514 | Outbreak 3 |
| 12-1886 | Food | PRJNA454819 | SAMN09668392 | Outbreak 3 |
| 12-1887 | Food | PRJNA454819 | SAMN09668393 | Outbreak 3 |
| 12-3021 | Human | PRJNA481261 | SAMN09738515 | Outbreak 4 |
| 12-3022 | Human | PRJNA481261 | SAMN09738516 | Outbreak 4 |
| 12-3034 | Human | PRJNA481261 | SAMN09738517 | Outbreak 4 |
| 12-3035 | Human | PRJNA481261 | SAMN09738518 | Outbreak 4 |
| 12-3036 | Human | PRJNA481261 | SAMN09738519 | Outbreak 4 |
| 12-3037 | Human | PRJNA481261 | SAMN09738520 | Outbreak 4 |
| 12-3038 | Human | PRJNA481261 | SAMN09738521 | Outbreak 4 |
| 12-3039 | Human | PRJNA481261 | SAMN09738522 | Outbreak 4 |
| 12-3062 | Human | PRJNA481261 | SAMN09738523 | Outbreak 4 |
| 12-3079 | Human | PRJNA481261 | SAMN09738524 | Outbreak 4 |
| 12-3080 | Human | PRJNA481261 | SAMN09738525 | Outbreak 4 |
| 12-3081 | Human | PRJNA481261 | SAMN09738526 | Outbreak 4 |
| 12-3082 | Human | PRJNA481261 | SAMN09738527 | Outbreak 4 |
| 12-3083 | Human | PRJNA481261 | SAMN09738528 | Outbreak 4 |
| 12-3084 | Human | PRJNA481261 | SAMN09738529 | Outbreak 4 |
| 12-3137 | Human | PRJNA481261 | SAMN09738530 | Outbreak 4 |
| 12-3138 | Human | PRJNA481261 | SAMN09738531 | Outbreak 4 |
| 12-3140 | Human | PRJNA481261 | SAMN09738532 | Outbreak 4 |
| 12-3212 | Human | PRJNA481261 | SAMN09738533 | Outbreak 4 |
| 12-3213 | Human | PRJNA481261 | SAMN09738534 | Outbreak 4 |
| 12-3303 | Human | PRJNA481261 | SAMN09738535 | Outbreak 4 |
| 12-3457 | Human | PRJNA481261 | SAMN09738537 | Outbreak 4 |
| 12-3474 | Human | PRJNA481261 | SAMN09738538 | Outbreak 4 |
| 12-6218 | Food | PRJNA454819 | SAMN09668394 | Outbreak 5 |
| 12-6416 | Human | PRJNA481261 | SAMN09738544 | Outbreak 5 |
| 12-6417 | Human | PRJNA481261 | SAMN09738545 | Outbreak 5 |
| 12-6418 | Human | PRJNA481261 | SAMN09738546 | Outbreak 5 |
| 12-6419 | Human | PRJNA481261 | SAMN09738547 | Outbreak 5 |
| 12-6569 | Food | PRJNA481261 | SAMN09738450 | Outbreak 5 |
| 12-6574 | Food | PRJNA454819 | SAMN09668395 | Outbreak 5 |
| 12-6575 | Food | PRJNA454819 | SAMN09668396 | Outbreak 5 |
| 12-6660 | Human | PRJNA481261 | SAMN09738550 | Outbreak 5 |
| 12-6794 | Human | PRJNA481261 | SAMN09738551 | Outbreak 5 |
| 12-6795 | Human | PRJNA481261 | SAMN09738552 | Outbreak 5 |
| 12-6797 | Human | PRJNA481261 | SAMN09738553 | Outbreak 5 |
| 12-6799 | Human | PRJNA481261 | SAMN09738554 | Outbreak 5 |
| 12-6801 | Human | PRJNA481261 | SAMN09738555 | Outbreak 5 |
| 12-6802 | Human | PRJNA481261 | SAMN09738556 | Outbreak 5 |
| 12-6845 | Human | PRJNA481261 | SAMN09738557 | Outbreak 5 |
| 12-6849 | Human | PRJNA481261 | SAMN09738558 | Outbreak 5 |
| 12-6956 | Human | PRJNA481261 | SAMN09738560 | Outbreak 5 |
| 12-6988 | Food | PRJNA481261 | SAMN09738451 | Outbreak 5 |
| 12-6991 | Human | PRJNA481261 | SAMN09738561 | Outbreak 5 |
| 12-7108 | Human | PRJNA481261 | SAMN09738564 | Outbreak 5 |
| 12-7322 | Food | PRJNA481261 | SAMN09738452 | Outbreak 5 |
| 12-7324 | Food | PRJNA481261 | SAMN09738453 | Outbreak 5 |
| 12-7387 | Human | PRJNA481261 | SAMN09738565 | Outbreak 5 |
| 12-7726 | Human | PRJNA481261 | SAMN09738568 | Outbreak 5 |
| 12-7002 | Human | PRJNA481261 | SAMN09738562 | Outbreak 6 |
| 12-7067 | Human | PRJNA481261 | SAMN09738563 | Outbreak 6 |
| 12-7659 | Human | PRJNA481261 | SAMN09738567 | Outbreak 6 |
| 12-7772 | Food | PRJNA481261 | SAMN09738454 | Outbreak 6 |
| 12-8044 | Human | PRJNA481261 | SAMN09738570 | Outbreak 6 |
| 12-8207 | Human | PRJNA481261 | SAMN09738571 | Outbreak 6 |
| 12-8354 | Food | PRJNA454819 | SAMN09668397 | Outbreak 6 |
| 12-8355 | Food | PRJNA454819 | SAMN09668398 | Outbreak 6 |
| 12-8356 | Food | PRJNA454819 | SAMN09668399 | Outbreak 6 |
| 12-8357 | Food | PRJNA454819 | SAMN09668400 | Outbreak 6 |
| 12-8358 | Food | PRJNA454819 | SAMN09668401 | Outbreak 6 |
| 12-8359 | Food | PRJNA454819 | SAMN09668402 | Outbreak 6 |
| 12-8360 | Food | PRJNA454819 | SAMN09668403 | Outbreak 6 |
| 12-8364 | Food | PRJNA454819 | SAMN09668404 | Outbreak 6 |
| 12-8422 | Food | PRJNA454819 | SAMN09668405 | Outbreak 6 |
| 12-8423 | Food | PRJNA454819 | SAMN09668406 | Outbreak 6 |
| 12-8424 | Food | PRJNA454819 | SAMN09668407 | Outbreak 6 |
| 12-8425 | Food | PRJNA454819 | SAMN09668408 | Outbreak 6 |
| 12-8426 | Food | PRJNA454819 | SAMN09668409 | Outbreak 6 |
| 12-8431 | Food | PRJNA454819 | SAMN09668410 | Outbreak 6 |
| 12-8485 | Food | PRJNA454819 | SAMN09668411 | Outbreak 6 |
| 12-8486 | Food | PRJNA454819 | SAMN09668412 | Outbreak 6 |
| 12-8594 | Human | PRJNA481261 | SAMN09738573 | Outbreak 6 |
| 13-0673 | Human | PRJNA481261 | SAMN09738605 | Outbreak 6 |
| 13-0812 | Human | PRJNA481261 | SAMN09738607 | Outbreak 6 |
| 13-0838 | Food | PRJNA481261 | SAMN09738455 | Outbreak 6 |
| 13-0897 | Food | PRJNA454819 | SAMN09668413 | Outbreak 6 |
| 13-0898 | Food | PRJNA454819 | SAMN09668414 | Outbreak 6 |
| 13-0899 | Food | PRJNA454819 | SAMN09668415 | Outbreak 6 |
| 13-0900 | Food | PRJNA454819 | SAMN09668416 | Outbreak 6 |
| 13-0980 | Food | PRJNA454819 | SAMN09668417 | Outbreak 6 |
| 13-0981 | Food | PRJNA454819 | SAMN09668418 | Outbreak 6 |
| 13-1020 | Human | PRJNA481261 | SAMN09738608 | Outbreak 6 |
| 13-1080 | Food | PRJNA454819 | SAMN09668419 | Outbreak 6 |
| 13-1183 | Food | PRJNA454819 | SAMN09668420 | Outbreak 6 |
| 13-1184 | Food | PRJNA454819 | SAMN09668421 | Outbreak 6 |
| 13-0015 | Human | PRJNA481261 | SAMN09738574 | Outbreak 7 |
| 13-0016 | Human | PRJNA481261 | SAMN09738575 | Outbreak 7 |
| 13-0017 | Human | PRJNA481261 | SAMN09738576 | Outbreak 7 |
| 13-0018 | Human | PRJNA481261 | SAMN09738577 | Outbreak 7 |
| 13-0019 | Human | PRJNA481261 | SAMN09738578 | Outbreak 7 |
| 13-0020 | Human | PRJNA481261 | SAMN09738579 | Outbreak 7 |
| 13-0021 | Human | PRJNA481261 | SAMN09738580 | Outbreak 7 |
| 13-0022 | Human | PRJNA481261 | SAMN09738581 | Outbreak 7 |
| 13-0023 | Human | PRJNA481261 | SAMN09738582 | Outbreak 7 |
| 13-0081 | Human | PRJNA481261 | SAMN09738583 | Outbreak 7 |
| 13-0082 | Human | PRJNA481261 | SAMN09738584 | Outbreak 7 |
| 13-0083 | Human | PRJNA481261 | SAMN09738585 | Outbreak 7 |
| 13-0085 | Human | PRJNA481261 | SAMN09738586 | Outbreak 7 |
| 13-0086 | Human | PRJNA481261 | SAMN09738587 | Outbreak 7 |
| 13-0087 | Human | PRJNA481261 | SAMN09738588 | Outbreak 7 |
| 13-0088 | Human | PRJNA481261 | SAMN09738589 | Outbreak 7 |
| 13-0137 | Human | PRJNA481261 | SAMN09738590 | Outbreak 7 |
| 13-0138 | Human | PRJNA481261 | SAMN09738591 | Outbreak 7 |
| 13-0140 | Human | PRJNA481261 | SAMN09738593 | Outbreak 7 |
| 13-0143 | Human | PRJNA481261 | SAMN09738594 | Outbreak 7 |
| 13-0145 | Human | PRJNA481261 | SAMN09738595 | Outbreak 7 |
| 13-0146 | Human | PRJNA481261 | SAMN09738596 | Outbreak 7 |
| 13-0224 | Human | PRJNA481261 | SAMN09738597 | Outbreak 7 |
| 13-0225 | Human | PRJNA481261 | SAMN09738598 | Outbreak 7 |
| 13-0226 | Human | PRJNA481261 | SAMN09738599 | Outbreak 7 |
| 13-0290 | Human | PRJNA481261 | SAMN09738600 | Outbreak 7 |
| 13-0292 | Human | PRJNA481261 | SAMN09738601 | Outbreak 7 |
| 13-0293 | Human | PRJNA481261 | SAMN09738602 | Outbreak 7 |
| 13-0294 | Human | PRJNA481261 | SAMN09738603 | Outbreak 7 |
| 13-0317 | Human | PRJNA481261 | SAMN09738604 | Outbreak 7 |
| 13-0696 | Human | PRJNA481261 | SAMN09738606 | Outbreak 7 |
| 13-4345 | Human | PRJNA481261 | SAMN09738610 | Outbreak 8 |
| 13-4436 | Human | PRJNA481261 | SAMN09738611 | Outbreak 8 |
| 13-4681 | Human | PRJNA481261 | SAMN09738612 | Outbreak 8 |
| 13-4819 | Human | PRJNA481261 | SAMN09738613 | Outbreak 8 |
| 13-5256 | Human | PRJNA481261 | SAMN09738616 | Outbreak 8 |
| 13-5288 | Human | PRJNA481261 | SAMN09738617 | Outbreak 8 |
| 13-5290 | Human | PRJNA481261 | SAMN09738618 | Outbreak 8 |
| 13-5291 | Human | PRJNA481261 | SAMN09738619 | Outbreak 8 |
| 13-5455 | Human | PRJNA481261 | SAMN09738620 | Outbreak 8 |
| 13-5456 | Human | PRJNA481261 | SAMN09738621 | Outbreak 8 |
| 13-5469 | Human | PRJNA481261 | SAMN09738622 | Outbreak 8 |
| 13-5474 | Human | PRJNA481261 | SAMN09738623 | Outbreak 8 |
| 13-5698 | Human | PRJNA481261 | SAMN09738624 | Outbreak 8 |
| 13-5719 | Human | PRJNA481261 | SAMN09738625 | Outbreak 8 |
| 13-5732 | Human | PRJNA481261 | SAMN09738626 | Outbreak 8 |
| 13-5754 | Human | PRJNA481261 | SAMN09738627 | Outbreak 8 |
| 13-5755 | Human | PRJNA481261 | SAMN09738628 | Outbreak 8 |
| 13-5758 | Human | PRJNA481261 | SAMN09738629 | Outbreak 8 |
| 13-5760 | Human | PRJNA481261 | SAMN09738631 | Outbreak 8 |
| 13-5761 | Human | PRJNA481261 | SAMN09738632 | Outbreak 8 |
| 13-5763 | Human | PRJNA481261 | SAMN09738633 | Outbreak 8 |
| 13-5807 | Human | PRJNA481261 | SAMN09738634 | Outbreak 8 |
| 13-5813 | Human | PRJNA481261 | SAMN09738635 | Outbreak 8 |
| 13-5819 | Food | PRJNA481261 | SAMN09738456 | Outbreak 8 |
| 13-5820 | Food | PRJNA481261 | SAMN09738457 | Outbreak 8 |
| 13-5821 | Food | PRJNA481261 | SAMN09738458 | Outbreak 8 |
| 13-5822 | Food | PRJNA481261 | SAMN09738459 | Outbreak 8 |
| 13-5823 | Food | PRJNA481261 | SAMN09738460 | Outbreak 8 |
| 13-5824 | Food | PRJNA481261 | SAMN09738461 | Outbreak 8 |
| 13-5825 | Food | PRJNA454819 | SAMN09668422 | Outbreak 8 |
| 13-5826 | Food | PRJNA454819 | SAMN09668423 | Outbreak 8 |
| 13-5827 | Food | PRJNA454819 | SAMN09668424 | Outbreak 8 |
| 13-5828 | Food | PRJNA481261 | SAMN09738462 | Outbreak 8 |
| 13-5830 | Food | PRJNA454819 | SAMN09668425 | Outbreak 8 |
| 13-5831 | Food | PRJNA454819 | SAMN09668426 | Outbreak 8 |
| 13-5930 | Food | PRJNA454819 | SAMN09668427 | Outbreak 8 |
| 13-5974 | Human | PRJNA481261 | SAMN09738637 | Outbreak 8 |
| 13-6007 | Human | PRJNA481261 | SAMN09738638 | Outbreak 8 |
| 13-6159 | Human | PRJNA481261 | SAMN09738639 | Outbreak 8 |
| 13-6160 | Human | PRJNA481261 | SAMN09738640 | Outbreak 8 |
| 13-6177 | Food | PRJNA454819 | SAMN09668428 | Outbreak 8 |
| 13-6178 | Food | PRJNA454819 | SAMN09668429 | Outbreak 8 |
| 13-6321 | Human | PRJNA481261 | SAMN09738641 | Outbreak 8 |
| 13-6444 | Human | PRJNA481261 | SAMN09738642 | Outbreak 8 |
| 11-1865 | Human | PRJNA481261 | SAMN09738476 | Sporadic |
| 11-2704 | Human | PRJNA481261 | SAMN09738480 | Sporadic |
| 11-2838 | Human | PRJNA481261 | SAMN09738482 | Sporadic |
| 11-3301 | Human | PRJNA481261 | SAMN09738483 | Sporadic |
| 11-3726 | Human | PRJNA481261 | SAMN09738484 | Sporadic |
| 11-4712 | Human | PRJNA481261 | SAMN09738485 | Sporadic |
| 11-5140 | Human | PRJNA481261 | SAMN09738486 | Sporadic |
| 11-5371 | Human | PRJNA481261 | SAMN09738487 | Sporadic |
| 11-5569 | Human | PRJNA481261 | SAMN09738489 | Sporadic |
| 11-5786 | Human | PRJNA481261 | SAMN09738490 | Sporadic |
| 11-5789 | Human | PRJNA481261 | SAMN09738491 | Sporadic |
| 11-6021 | Human | PRJNA481261 | SAMN09738492 | Sporadic |
| 11-6913 | Human | PRJNA481261 | SAMN09738497 | Sporadic |
| 11-7032 | Human | PRJNA481261 | SAMN09738498 | Sporadic |
| 11-7132 | Human | PRJNA481261 | SAMN09738499 | Sporadic |
| 11-7301 | Human | PRJNA481261 | SAMN09738500 | Sporadic |
| 11-7639 | Human | PRJNA481261 | SAMN09738504 | Sporadic |
| 12-0104 | Human | PRJNA481261 | SAMN09738508 | Sporadic |
| 12-0356 | Human | PRJNA481261 | SAMN09738510 | Sporadic |
| 12-1789 | Human | PRJNA481261 | SAMN09738513 | Sporadic |
| 12-2955 | Environmental | PRJNA481261 | SAMN09738448 | Sporadic |
| 12-3387 | Human | PRJNA481261 | SAMN09738536 | Sporadic |
| 12-4277 | Human | PRJNA481261 | SAMN09738539 | Sporadic |
| 12-4306 | Human | PRJNA481261 | SAMN09738540 | Sporadic |
| 12-5469 | Human | PRJNA481261 | SAMN09738541 | Sporadic |
| 12-5484 | Human | PRJNA481261 | SAMN09738542 | Sporadic |
| 12-6061 | Human | PRJNA481261 | SAMN09738543 | Sporadic |
| 12-6581 | Human | PRJNA481261 | SAMN09738548 | Sporadic |
| 12-6654 | Human | PRJNA481261 | SAMN09738549 | Sporadic |
| 12-6887 | Human | PRJNA481261 | SAMN09738559 | Sporadic |
| 12-7435 | Human | PRJNA481261 | SAMN09738566 | Sporadic |
| 12-7844 | Human | PRJNA481261 | SAMN09738569 | Sporadic |
| 12-8208 | Human | PRJNA481261 | SAMN09738572 | Sporadic |
| 13-0139 | Human | PRJNA481261 | SAMN09738592 | Sporadic |
| 13-3867 | Human | PRJNA481261 | SAMN09738609 | Sporadic |
| 13-4894 | Human | PRJNA481261 | SAMN09738614 | Sporadic |
| 13-4900 | Human | PRJNA481261 | SAMN09738615 | Sporadic |
| 13-5759 | Human | PRJNA481261 | SAMN09738630 | Sporadic |
| 13-5839 | Human | PRJNA481261 | SAMN09738636 | Sporadic |
| 13-7130 | Human | PRJNA481261 | SAMN09738643 | Sporadic |
